# Prevalence of overweight, obesity and central obesity and factors associated with BMI in indigenous yaqui people: a probabilistic cross-sectional survey

**DOI:** 10.1186/s12889-022-12702-2

**Published:** 2022-02-14

**Authors:** Araceli Serna-Gutiérrez, Alejandro Arturo Castro-Juarez, Martín Romero-Martínez, Heliodoro Alemán-Mateo, Rolando Giovanni Díaz-Zavala, Luis Quihui-Cota, Gerardo Álvarez-Hernández, Ana Cristina Gallegos-Aguilar, Julián Esparza-Romero

**Affiliations:** 1 Sociocultural Department, Technological Institute of Sonora, 85137 Cd. Obregón, Sonora, México; 2 Diabetes Research Unit, Department of Public Nutrition and Health, Nutrition Coordination, Research Center for Food and Development (CIAD, A.C.), 83304 Hermosillo, Sonora, México; 3 Evaluation and Surveys Research Center, National Institute of Public Health, 62100 Cuernavaca, Morelos, México; 4 Department of Nutrition and Metabolism, Nutrition Coordination, Research Center for Food and Development (CIAD. A.C.), 83304 Hermosillo, Sonora, México; 5 Nutrition Health Promotion Center, Department of Chemical and Biological Sciences, University of Sonora, 83000 Hermosillo, Sonora, México; 6 Department of Medicine and Health Sciences, University of Sonora, 83000 Hermosillo, Sonora, México; 7 Diabetes Research Unit, Deparment of Public Nutrition and Health, Nutrition Coordination, Research Center for Food and Development (CIAD, A.C.), Carretera Gustavo Enrique Astiazarán Rosas No. 46, Col. La Victoria, 83304 Hermosillo, Sonora, México

**Keywords:** Indigenous Yaqui, Prevalence, Overweight, Obesity, Central obesity, Survey, Associated factors, México

## Abstract

**Background:**

The Yaquis are an Indigenous group who inhabit in the state of Sonora in northwestern Mexico. This group has experienced changes in their lifestyle, moving from a traditional lifestyle to a more modern one, resulting in an increase of obesity and its comorbidities. However, few studies have been done in this group. The aim of this study was to determine the prevalence of overweight, obesity and central obesity and to identify the factors associated with body mass index (BMI) in a representative sample of Indigenous Yaqui people from Sonora, Mexico.

**Methods:**

A cross-sectional survey with multistage sampling was conducted among adults (*N* = 351) with residence in Yaqui traditional villages (Vícam, Pótam, Loma de Guamúchil, Loma de Bácum, Tórim, Ráhum, Huiribis or Belem). Anthropometric measurements were taken to diagnose overweight, obesity and central obesity. Food frequency and physical activity (PA) questionnaires designed for the Yaqui population were applied, as well as sociodemographic and clinical history questionnaires. The factors associated with BMI were assessed using multiple linear regression considering the complex design of the sampling.

**Results:**

The prevalence of overweight, obesity and central obesity in the population were 36.5%, 35.0% and 76.0%, respectively. Having higher values of the modernization index (β = 0.20, *p* = 0.049) was associated with a higher BMI, while having a higher consumption of a “prudent” dietary pattern (traditional dishes, fruits, vegetables and low-fat dairy) (β = -0.58, *p* = 0.009) and performing a greater number of hours per week of vigorous PA (β = -0.14, *p* = 0.017) were associated with a lower BMI.

**Conclusions:**

The prevalence of the studied abnormalities is high. The evidence presented in this study suggests that interventions are needed and more research is required to determine the appropriate components of such interventions, in order to meet the needs of the Yaqui people.

## Background

Overweight and obesity are serious health problems worldwide including in Indigenous groups [1, 2]. According to the Global Burden of Disease Study in 2017, 4.7 million deaths and 148 million disability-adjusted life years were attributed to a higher BMI [3]. In addition, obesity is the main modifiable risk factor for the development of noncommunicable diseases (NCDs), such as type 2 diabetes (T2D) and cardiovascular disease (CVD), pathologies that are considered pandemic [4]. In Mexico, T2D and CVD are the leading causes of death and generate excessive health expenses [5]. In addition, obesity is associated with an increased risk of severe disease [6] and mortality [7] from COVID-19, a disease that has caused more than 233,950 deaths in Mexico to date [8].

The origins of obesity are multifactorial, and its development is related to lifestyle (unhealthy diet and low level of physical activity) [9, 10], genetics [11], sociodemographic variables (sex and age) [12], economic condition [13], education [14] and environmental factors [15, 16]. Indigenous groups have greater poverty, less schooling, less access to health services and some are modifying their lifestyle, which consequently makes them extremely vulnerable to obesity [2, 17, 18].

The study of prevalence and the identification of factors associated with obesity in population groups are necessary for the design of adequate prevention programs, especially in communities lacking in medical services, such as Indigenous localities [2]. In Mexico, national health surveys provide information on the prevalence of overweight/obesity for the general population [19]; however, the prevalence and risk factors for obesity in Mexican Indigenous groups are unknown. Regarding to the factors associated with obesity, information is missing or scarce for many Indigenous peoples [20], despite its high prevalence in no representative studies [21–24]. It is imperative that epidemiological data are generated to guide public policies.

The Yaquis are an Indigenous group who inhabit the center-south region of the state of Sonora in northwestern Mexico and are mainly distributed in eight traditional villages [25]. This group has experienced changes in their lifestyle, moving from a traditional lifestyle to a more modern one, which is characterized by a decrease in agricultural activities and an increase in a sedentary lifestyle [26]. It has also been reported that the tradition of family gardens has been lost [27] and that processed foods are highly available [26]. As a result, this Indigenous group has serious problems with diseases associated with obesity such as T2D  [24] and hypertension [28].

Previous studies have documented a high prevalence of obesity in the Yaquis [23, 24, 26]. However, such studies have not been done in a way that represents the eight traditional Yaqui towns. Furthermore, no study has considered probabilistic sampling to estimate the prevalence of obesity and its associated factors in this Indigenous group. The purposes of this work were (1) to examine the current prevalence of overweight, obesity and central obesity in the total population and by sex, and (2) to identify factors associated with the BMI of Indigenous Yaqui adults in a probabilistic sample representative of the adult Yaqui natives who live in the eight traditional villages in Sonora, Mexico.

## Methods

 The current cross-sectional survey design was conducted from May to September 2017 and was approved by the ethics committee of the Centro de Investigación en Alimentación y Desarrollo (CE/007/2016). The sample size was determined as indicated by Romero et al. [29]. The estimated sample size was 294 households, which was rounded to 351 to compensate for a potentially low participation rate and to obtain statistical power.

The sampling frame was made up of a list of blocks from the 2010 Population and Housing Census for the Yaqui urban communities (Vícam and Pótam)[30] and a list of blocks generated by the working group for the Yaqui rural communities (Loma de Guamúchil, Loma de Bácum, Tórim, Ráhum, Huiribis and Belem)[30]. The target population was Yaqui adults, men and women, age ≥ 20 years old.

A multistage probabilistic sampling design stratified by the urban and rural communities was used. In the first stage of sampling, the selected blocks were visited in each town. In urban localities, conglomerates (blocks) were randomly selected with probabilities proportional to their size (number of dwellings). In rural communities, because the generated blocks were similar in number of dwellings (approximately 15), block selection was made in a simple random manner. In the second stage, after listing the houses in the selected blocks (from urban and rural locations), 12 or more households per block were selected by systematic selection. In the final stage, one participant was chosen per household using the Kish [31] probabilistic method. In each of the villages, the survey was carried out with the support of a Yaqui resident health assistant.

People aged <20 years, those who did not belong to the Yaqui ethnic group, those who were pregnant or lactating women, bedridden patients, people who were mentally ill and people with walking disabilities were considered ineligible. To ensure that the selected population was of Yaqui origin, an adult living in the selected household was interviewed about their ethnic origin. Those who descended from Yaqui parents or grandparents were considered to be of Yaqui origin [32].

Once the selected subject was identified, both the study protocol and the requirements necessary for the study were explained. Those who wished to participate signed a consent form. The measurements were made at the household of the selected people as scheduled. If the selected person did not choose to participate, a new home, located to the right of the original home was then selected. To estimate the percent of participation, people who refused to be evaluated or were not found at home on the day of the evaluation were considered nonparticipants.

### Anthropometric and physical measurements

Weight, height and waist circumference were measured by trained personnel using the methodology of the International Association of Cineanthropometry [33]. From this information, the BMI was calculated. The weight and height were measured using a SECA 813 electronic scale with a capacity of 200 kg ± 100 g and a SECA 213 stadiometer with a precision of 1 mm. The waist circumference was measured using a Gülick fiberglass tape. Systolic and diastolic blood pressure was determined with the average of two measurements using an Omron HEM-907.XL blood pressure monitor following the protocol of the American Heart Association [34]. Overweight and obesity were diagnosed as recommended by the World Health Organization [35] and central obesity as recommended by the International Diabetes Federation [36].

### Evaluation of diet

The dietary information was obtained through a food frequency questionnaire (FFQ) designed for Indigenous Yaqui people that included 123 foods [37]. The average daily consumption of energy and macronutrients was estimated using a food composition database [38].

To obtain the dietary patterns, the principal component (PC) technique was used. In the first step, daily consumption in grams of each of the 123 foods was calculated for each participant. Then, a univariate analysis of each food was performed with the response variable (BMI). In the second step, foods showing a plausible association with BMI were classified into 18 food groups, as reported by Newby et al. [39]. A new dataset was then generated with the amount (g/d) of consumption of each food group per participant. The adequacy of this new dataset was assessed by the Kaiser-Meyer-Olkin test (KMO> 0.6). In the third step, the PC technique and an orthogonal rotation were performed to determine the factorial loads of the 18 food groups. The number of dietary patterns identified was based on eigenvalues ≥1.5, identification of the inflection point in the scree plot and interpretability. Moreover, food groups with a load factor ≥ | 0.3 | were considered significant contributors to the selected component [40]. Derived patterns were labeled based on the food groups they contained, as in previous studies [39].

### Evaluation of physical activity

Physical activity (PA) was evaluated with a questionnaire adapted for adult Yaquis [41]. The questionnaire was used to collect information from the previous 12 months on the frequency and duration of occupational, leisure time PA (light, moderate and vigorous) and sedentary habits. Finally, the PA was expressed in hours per week (h/week) considering the intensity [42].

### Sociodemographic and clinical history questionnaires

Sociodemographic variables were collected with a sociodemographic questionnaire (SQ) that was designed considering an SQ used with Pima Indigenous people [43] and the household questionnaire of the National Health and Nutrition Survey of Midway 2016 (ENSANUT MC 2016) [44]. For the SQ, the most relevant questions were selected and adapted for the study population. Sociodemographic variables included sex, age, marital status, educational level, Yaqui language, and number of household members, among others. Regarding the socioeconomic characteristics, the SQ also included questions about home, automobile, motorcycle, farm machinery and farmland ownership, as well as employment, housing characteristics and others.

The SQ allowed the generation of the modernization index (MI) variable by asking the participant about different technological assets in their home (television, refrigerator, microwave oven, stove, washing machine, air conditioning, computer, satellite signal, etc.). This variable allowed the estimation of the degree of modern lifestyle of the participants.

A clinical history questionnaire was designed based on the adult questionnaire of the National Health and Nutrition Survey of 2006 [45] and the history questionnaire on chronic diseases [44] of the ENSANUT MC 2016. The questionnaire was applied to obtain basic information about the participant’s health status. Questions about obesity related diseases and their treatments, habits of smoking and alcohol consumption were also included.

### Statistical analysis

The selected sample was weighted to take into account the unequal selection probabilities due to the complex sampling design. For the calculation of the weights, the inverse of the selection probability, the response rate and a calibration factor were considered. In addition, information about the sampling design, the stratum, the primary sampling unit (blocks) and the weights were assigned to each participant [46]. The analysis was performed using Stata, version 14.0 (StataCorp) and adjusted to the sampling design of the survey using the svy command.

The means and proportions of sociodemographic, clinical, dietary and physical characteristics were calculated, and the Wald chi square and t tests as required, were used to compare these characteristics between men and women. The prevalence of overweight, obesity and central obesity and the 95% confidence intervals were determined for the total population and for each sex, adjusted for age. For the latter, the direct standardization method was considered, using the population analyzed as the standard population. The prevalence described above was also estimated by age category, MI (tertiles), marital status and other social and health related variables. A Wald chi square test was used to compare the prevalence by category of the indicated variables.

To identify factors associated with the BMI, a weighted multiple linear regression analysis considering the complex design of the survey was performed. For the generation of the predictive preliminary model of multiple linear regression, a mixture of univariate and stepwise analysis was used. All variables (social, clinical history and lifestyle) were analyzed by simple linear regression and variables with a value of *p* ≤ 0·2 and biological plausibility were considered as possible predictors and/or adjusted variables in the stepwise model selection (*p* ≤ 0·05). The preliminary model was evaluated for the presence of interaction (*P* ≤ 0 ·1) and collinearity (VIF > 10), as well as linear regression assumptions.

## Results

A total of 470 Yaqui adults were interviewed. From these, 73 chose not to participate, 43 could not be evaluated and three were excluded for incomplete information (75.3% participation). Considering one adult per household, the final sample included 351 subjects, representing 6,887 Yaqui adults of 20 years of age or older; 202 women and 149 men, representing 3,597 women and 3,290 men, respectively.

The characteristics of the participants are indicated in Table 1. Significant differences were observed between sex categories. BMI was higher in women (*p* = 0.0001), while systolic blood pressure (*p* = 0.0094) and energy consumption (*p* = 0.0001) were higher in men. Regarding PA, compared to men, women performed less than half of the hours per week of moderate PA (*p* = 0.0001). In relation to vigorous PA, the difference was much larger, with many fewer hours per week in women (0.10 h/week) than in men (3.06 h/week) (*p* = 0.0001). The opposite was observed for light PA; the women participated in three times more h/week of light PA than men (*p* = 0.0001). No differences were observed in sedentary habits. On the other hand, MI (*p* = 0.043), smoking habits (*p* = 0.0001), and alcohol consumption were higher in men (*p* = 0.0001).

**Table 1:** Characteristics according to gender of Yaqui adults from Sonora, México^a^

Characteristics	Total	IC 95%	Women *n *= 202 (52%)	IC 95%	Men *n* = 149 (48%)	IC 95%	*p-*value^b^
Age (years)	40.7	38.5-42.2	42.0	39.4-44.5	39.2	36.2-42.1	0.0950
Weight (kg)	75.6	73.7-77.5	73.3	70.8-75.7	78.1	75.9-80.5	0.0075
Height (cm)	163.2	161.9 164.5	156.5	15.6-157.4	170.6	169.4-171.8	0.0001
BMI (kg/m^2^)	28.4	27.7-29.0	29.8	28.9-30.8	26.8	26.0-27.5	0.0001
Waist Circumference (cm)	95.1	93.3-96.8	95.4	95.5-98.3	94.7	93.0-96.4	0.6314
Systolic blood pressure (mmHg)	118.0	115.8-120.2	114.7	114.4-117.9	121.6	118.5-124.8	0.0094
Diastolic blood pressure (mmHg)	73.4	72.5-74.4	72.5	70.9-74.1	74.5	72.6-76.3	0.1741
Energy diet (kcal/day)	3076	2851-3301	2734	2467-3001	3449	3158-3741	0.0001
Energy from carbohydrate (%)	57.1	56.3-58.0	57.3	56.4-58.3	56.9	55.6-58.3	0.6505
Energy from fat (%)	30.8	30.0-31.5	30.4	29.5-31.3	31.2	29.9-32.4	0.2984
Energy from protein (%)	12.0	11.7-12.3	12.2	11.8-12.5	11.7	11.2-2.2	0.1718
Physical activity							
Light (h/wk)	14.6	12.4-16.8	21.4	18.5-24.3	7.2	4.6-9.7	0.0001
Moderate (h/wk)	14.3	12.3-16.3	9.3	7.1-11.6	19.8	16.6-22.9	0.0001
Vigorous (h/wk)	1.5	1.0-2.0	0.1	0.02-0.1	3	1.9-4.1	0.0001
Sedentary habits (h/wk)	15.2	13.6-16.7	15.2	12.8-17.7	15.1	13.3-16.9	0.9480
Modernization index	8.9	8.4-9.4	8.4	7.8-9.0	9.4	8.7-10.1	0.0430
Location							0.5222
Rural (%)	35.1	21.5-48.7	36.8	22.0-51.7	33.2	16.6-49.7	
Urban (%)	64.9	51.2-78.4	63.1	48.2-77.9	66.7	50.2-83.3	
Speak Yaqui language (%)	70.9	61.3-80.5	73.3	61.8-84.8	68.3	55.0-81.5	0.5072
Education level							0.2846
Less than primary (%)	27.2	21.2-33.1	25.6	18.0-35.0	28.9	20.3-39.4	
Primary (%)	17.9	13.1-22.7	16.3	23.5	19.8	14.6-26.9	
Basic (%)	33.4	26.1-40.8	31.4	23.4-40.6	35.7	24.7-49.0	
High school (%)	16.4	11.2-21.6	19.3	13.5-26.7	13.3	6.9-24.0	
More than high school (%)	4.8	2.1-7.5	7.3	4.2-12.3	2.1	0.6-6.4	
Employment situation							0.0001
Housewife (%)	29.9	24.3-35.5	57.0	47-66.5	0.00	0.00	
Permanent employment (%)	25.4	206-30.3	17.6	11.8-25.3	34.1	26.2-43.0	
Temporal employment (%)	21.3	15.1-27.5	10.4	5.5-18.7	33.2	24.1-43.7	
Self-employment (%)	15.3	11.6-19.1	11.9	7.5-18.1	19.2	11.8-29.6	
Unemployed (%)	6.2	3.0-9.4	2.5	0.9-6.2	10.5	5.9-18.2	
Student (%)	1.5	10.4-3.6	0.5	0.0-3.6	2.8	0.0-11.2	
Marital status							0.1508
Single/divorced/widowed (%)	31.8	23.8-39.8	26.8	17.4-38.9	37.3	27.4-48.3	
Married/living with a partner (%)	68.1	60.1-76.1	73.12	61.1-82.5	62.7	51.6-72.5	
Current smoker (yes)	25.3	18.3-32.3	3.7	0.1-7.2	49.0	39.3-58.7	0.0001
Current alcohol consumer (yes)	43.3	34.8-51.8	23.0	14.4-31.6	65.4	52.4-78.4	0.0001

^a^ Data adjusted for the complex survey design. Values expressed as means (95%) o percentage (confidence interval at 95%)

^b^ Column comparison (women and men)

**Fig. 1:**
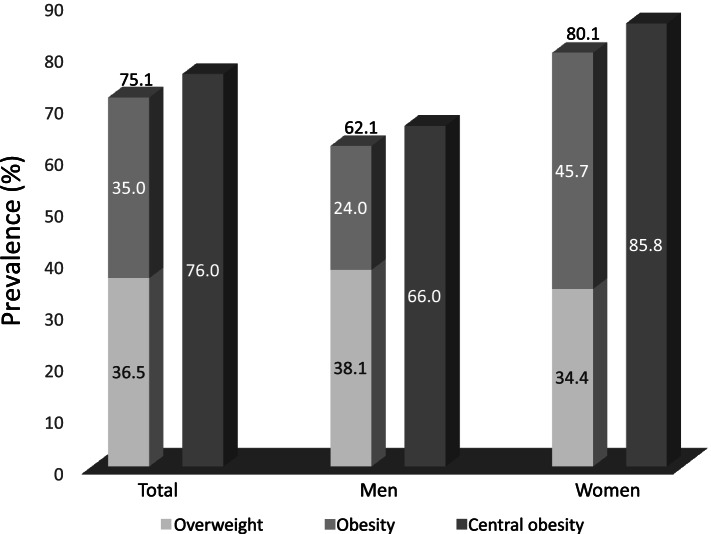
Prevalence of overweight, obesity and central obesity in Yaquis from Sonora, Mexico. * Data adjusted for the complex survey design. † Data adjusted for age, using the direct method of standardization

The combined prevalence of overweight/obesity in Yaqui adults of the state of Sonora was 71.5% (95% CI 65.9, 76.9) (representing 4,991 men and women of this age group) (Fig. 1). The prevalence of obesity was 35% (95% CI 28.7, 41.1) (2,409 people), while it was 36.5% for overweight (95% CI 29.1, 43.7) (2,512 people). By sex, the combined prevalence of overweight/obesity was higher in women (80.1%, 95% CI 73.0, 87.2) (2,876 women) than in men (62.1%, 95% CI 53.1, 71.0) (2,045 men). The prevalence of obesity was also higher in women (45.7%, 95% CI 37.6, 53.8) than in men (24%, 95% CI 15.4, 32.5), while overweight was higher in men than in women (38.1%, 95% CI 25.8, 50.3 and 34.4%, 95% CI 26.1, 42.6, respectively). Figure 1 shows the prevalence of central obesity, which was 76.0% (95% CI 71.1, 81.0) (representing 5,239 Yaqui adults). For men, the prevalence of central obesity was 66.0% (95% CI 57.3, 74.7) (2,151 men), while for women the prevalence was higher with a value of 85.8% (95% CI 79.08, 92.6) (3,088 women).

Table 2 shows the prevalence of overweight, obesity and central obesity according to different characteristics of the population. It should be considered that the sample sizes in each category of the characteristics shown are low, which explains why no significant differences are obtained in the same cases. However, the information in this regard is considered an important contribution in this Indigenous group. For instance, the age group with the highest prevalence of obesity in Yaqui adults was 30 to 39 years old, while those over 60 had the highest prevalence of overweight. Regarding the prevalence of central obesity, the highest prevalence occurred in the age range of 50 to 59 years old. However, the differences in the prevalence studied by age group were not significant.

**Table 2:** Prevalence of overweight, obesity and central obesity according to different characteristics^a^

Characteristics	Total		Overweight		Obesity		Central obesity	
	**n**	**N**	**%**	**IC 95%**	**%**	**IC 95%**	**%**	**IC 95%**
Age group (years)								
20-29	82	1931	35·9	20·2-51·6	26·9	14·9-39·0	64·5	46·3-82·7
30-39	90	1491	19·5	11·0-28·0	48·2	35·9-60·5	74·2	62·7-85·6
40-49	70	1410	36·5	17·2-55·7	43·1	25·8-60·5	83·0	71·9-94·0
50-59	59	1113	48·6	31·5-65·6	32·9	16·5-49·4	84·0	73·6-94·9
≥60	50	942	49·9	36·0-63·8	20·4	9·3-31·5	82·9	57·3-94·6
Location								
Rural	111	2410	36·6	28·1-45·0	35·4	27·0-43·8	70·3	62·6-78·1
Urban	240	4467	36·1	22·7-49·6	34·1	24·1-44·0	79·1	72·8-85·5
Education level								
Less than primary	100	1876	43·4	27·6-59·1	34·5	20·7-48·4	85·7	75·4-92·1
Primary	69	1238	28·3	16·4-40·2	33·3	18·8-47·8	64·4	49·5-77·0
Basic	109	2306	34·8	20·3-49·2	32·3	18·4-46·2	70·4	62·2-77·5
High school	54	1134	40·1	25·3-54·8	37·3	17·5-57·0	80	65·7-90·2
More than high school	19	333	26·8	3·0-50·6	53·1	26·9-79·3	87·4	51·3-97·8
Marital status								
Single/divorced/widowed	102	2194	36·8	25·2-48·3	18·2^a^	8·0-27·6	63·0^a^	49·8-76·1
Married/living with partner	249	4693	36·3	28·0-44·5	42·7^b^	32·7-52·8	82·1^b^	75·6-88·7
Modernization index								
Tertile 1	138	2469	41·1	29·7-52·4	31·7	22·1-41·2	75·3	65·8-84·7
Tertile 2	106	1876	36·3	21·5-51·1	30·5	17·5-43·5	76·3	69·0-83·6
Tertile 3	107	2542	32·0	19·5-44·6	41·3	29·6-53·1	76·6	67·7-85·4
Employment situation								
Housewife	113	2052	41·4	30·6-53	39·3	26·4-53·9	86·0	75·0-92·6
Permanent employment	78	1756	26·4	16·1-40·1	44·4	30·0-59·96	74·4	57·2-86·3
Temporal employment	79	1470	41·4	24·7-60·4	27·3	15·3-43·7	71·6	58·3-81·9
Self-employment	54	1061	38·7	25·0-54·4	29·4	15·8-48·1	72·4	55·9-84·5
Unemployed	24	438	19·4	73·0-42·0	24·2	9·7-48·7	57·4	36·4-76·0
Student	3	110	84·4	30·5-89·5	0	0	84·4	30·5-98·5
Current smoker								
Yes	77	1748	30·3	17·2-42·4	24·6	10·8-38·4	59·0^a^	47·1-70·9
No	274	5139	38·5	32·0-45·5	38·4	31·2-45·7	81·8^b^	75·7-87·9
Current alcohol consumer								
Yes	143	2985	36·0	27·3-44·8	32·6	22·6-42·5	74·4	65·4-83·4
No	208	3902	36·7	28·8-45·4	36·7	28·1-45·4	77·3	70·2-84·4

^a^Data adjusted for the complex survey design. Values expressed as percentage (confidence interval at 95%). n: sample studied, N: population that represents. Only where different letters are indicated p < 0.05, comparison between rows (by category of each variable)

Furthermore, no differences were found in prevalence by location (rural or urban), schooling, tertile of MI and employment situation (Table 2). Regarding the latter, when comparing the housewife category with the rest of the grouped categories, the people in charge of the housework presented a higher prevalence of central obesity (86%) than those who do not work in the household (71.4%) (*p* = 0.0419). Adults married or living with a partner had a higher prevalence of obesity and central obesity (*p* = 0.0075 and *p* = 0.0206, respectively) than single, divorced or widowed persons.

On the other hand, two dietary patterns were derived and labeled as follows: (1) Prudent pattern (high in traditional dishes, fruits, vegetables and low-fat dairy), and (2) Westernized pattern (high in eggs, processed meats and dressings). The factorial load of these dietary patterns explained 25% of the total variability in diet of the Yaqui adult population (Table 3).

**Table 3:** Factor load of food groups in dietary patterns

Food groups	Dietary Patterns	
	**Prudent**	**Westernized**
	Factor Load	
Traditional dishes	0.4409	-
Fruits	0.4264	-
Vegetables	0.3950	-
Low-fat dairy	0.3806	-
Eggs	-	0.5192
Dressing	-	0.4813
Processed meat	-	0.4445
Eigenvalue	2.9	1.5
Variance explained (%)	14.5	11.0

Factor load values <| 0.3 | were excluded for simplicity

In the adjusted multiple linear regression model, a lifestyle related factor that was associated with higher BMI was having higher values of MI (β = 0.20, *p* = 0.049). On the other hand, lifestyle related factors that were associated with a lower BMI were having a habit of a higher consumption of a “prudent” dietary pattern (β = -0.58, *p* = 0.009), as well as having a pattern with higher values of vigorous PA (β = -0.14, *p* = 0.017). All variables were adjusted for themselves and by age, sex, and marital status (Table 4).

**Table 4:** Main factors associated with BMI in Yaqui adults from Sonora, México^a^

Factors	Beta (β*)*	*p-*value
Modernization Index (score)	0.20	0.049
“Prudent” dietary pattern (score)	-0.58	0.009
Vigorous physical activity (h/wk)	-0.14	0.017

^a^ Data adjusted to the complex design of the survey. All the variables in the table were adjusted by themselves and by age, sex and marital status

## Discussion

The aim of this survey was to estimate the prevalence of obesity and the associated factors related with the BMI of Yaqui adults that inhabit the eight traditional villages of the tribe. To our knowledge, this is the first study in Mexico that has reported on the problem of obesity in an Indigenous group with the population size of the Yaquis using a representative sample and a probabilistic sampling design, which allowed valid inferences from the studied population. In terms of public policies, the study provides reliable and specific information to justify the development of programs aimed at combating obesity in this Indigenous group.

The observed overweight/obesity prevalence (71.5%) in Indigenous Yaquis is a call to public health action, since its high prevalence and because both conditions constitute main risk factors for T2D and CVD development. This prevalence is slightly lower to that reported at the national level (75.2%) [47]. This pattern is different from that in developed countries, where the prevalence of obesity is higher in Indigenous groups than in nonindigenous groups [48, 49]. However, this does not mean that the prevalence obtained is low; on the opposite, its high value is worrisome, and should be considered among the highest reported prevalence. When compared with the Pima Indigenous people from the state of Sonora (64.1%) [17], the prevalence of overweight/obesity in Yaqui adults is higher, and it is also well above than the rate reported for the Tojolabal Indigenous population from Comitán, Chiapas (48.7%) in southeastern Mexico [50].

The pattern of a much higher proportion of obesity in women than men (45.7% vs. 24.0%, respectively) may be explained to differences in PA, since women spend less h/week participating in moderate PA. This difference is also observed in the overall Mexican adult population [47] and in the Pima Indigenous people from Sonora [17]. In particular, Yaqui women have a higher prevalence of obesity (45.7%) than women at the national level [47] and Mexican Pima women [17], which suggests the need to design specific interventions for this population. The prevalence of central obesity for Yaqui population [76.0%] is slightly lower to that reported at the national level (81.6%) as a whole and by sex [47].

Considering the conditions of inequity in health status in Indigenous populations worldwide [2] and in Mexico [18], along with the high prevalence of overweight/obesity among Yaqui adults, the risk of suffering NCDs such as T2D and CVD in this ethnic group are much higher than in the nonindigenous population of Mexico. Furthermore, the high prevalence of overweight/obesity and central obesity in this Indigenous group implies that prevention and control actions for these conditions by health institutions are insufficient [51].

Regarding the factors associated with obesity in Yaqui adults, a higher MI value, which is based on technological home goods, was positively associated with the BMI in the studied population. Esparza et al. [17] discussed that MI represents an indicator of socioeconomic and behavioral factors that cause subtle changes in diet or energy expenditure in the Pima Indigenous people. Cassel mentions that modernity is associated with physical inactivity and a greater availability of energy-dense Western foods, increasing the risk of obesity [52]. Ther efore, it is possible that in Yaqui communities, those with better purchasing power, reflected in the MI, may also be more able to obtain densely energetic foods with low nutritional value [53]. For example, in the study population, the MI showed a positive association with the consumption of sugar-sweetened carbonated beverages (β = 0.0014, *p* = 0.002). On the other hand, greater than 50% of the study subjects own kitchen appliances that allow them to keep more food, so the family diet can be enriched. In addition, the use of appliances such as a washing machine could reduce energy expenditure. Likewise, having a television may be causing the increase in hours per week of sedentary habits. The results obtained agree with the study carried out in Maycoba, Sonora, Mexico, where in a population composed of Pima and non-Pima Indigenous individuals, the MI was associated with a higher BMI (β = 1.0, *p* = <0.0001) [17].

This is the first study that assessed dietary patterns in an Indigenous group in Mexico and its association with BMI. In the determination of dietary patterns, the dietary intake was not studied by the consumption of individual nutrients but by the complete dietary pattern, which more closely resembles the actual consumption of food by a person, that is, the effect of diet goes beyond individual nutrients and foods [54, 55]. In this study, a higher consumption of a prudent dietary pattern was associated with a decrease in BMI, which may indicate that people with a greater consumption of a prudent pattern characterized by traditional dishes (“gallina pinta” [broth of beans, corn, and beef], broth of cheese, “caldillo de machaca” [broth of dried beef and potatoes] and meatballs), fruits, vegetables and low-fat dairy (low-fat milk and fresh cheese) presented a protective effect regarding an increase in BMI.

Even though the traditional dishes include beef [with the exception of cheese broth], which has fat in its nutritional composition, the amount it contains explains why the Mexican System of Equivalent Food [56] classifies these foods as having a low to moderate contribution of fat. In addition, these dishes contain some vegetables, corn, beans and potatoes [57]. The prudent pattern also includes fruits and vegetables, which have low energy density and whose consumption could decrease the intake of foods rich in fats and sugars. In fact, the consumption of fruits and vegetables has been inversely associated with weight gain [58]. The prudent pattern also contains low-fat dairy products such as fresh cheese and skim milk; the latter is recommended over the consumption of whole milk to prevent the development of obesity [59]. The information obtained about this dietary pattern in the Yaquis is extremely important because it promotes the consumption of their traditional foods, in addition to fruits, vegetables and low-fat dairy, over processed foods to prevent obesity. Health campaigns and other state programs should consider this finding to create better dietary and nutritional interventions for obesity prevention in this Indigenous group.

The results regarding the prudent dietary pattern are consistent with previous studies in nonindigenous populations [60, 61]. In Mexico, a study conducted in a cohort of health workers reported an inverse relationship between a prudent pattern (high in fruits, vegetables and legumes and low in pies, refined cereals and cookies) and the risk of developing obesity and central obesity; however, the association was not significant [62]. On the other hand, some studies carried out on non-representative samples of Yaquis adults have also indicated a high consumption of calorie-dense foods as processed products, due to greater availability, since their communities are relatively close and with easy access to nearby cities [26, 37]. Likewise, a study conducted more than a decade ago in three traditional Yaqui localities reported a positive and significant association between saturated fat consumption and the prevalence of obesity [24].

Some studies have indicated that the Yaquis have decreased their agricultural related activities, moving to a more sedentary new occupational activities such as those associated with maquiladora companies [26, 41], which could aggravate the obesity problem. However, in the present study, total vigorous PA was a protective factor against an increase in BMI in the Yaqui adult population, and this mainly involved from work activities such as agriculture. Although the second source of work in the population is the maquiladora companies, the Yaqui men are also employed in the traditional manufacture of coal, firewood recollection and masonry, all activities of vigorous intensity. Some women are employed in weeding activities in agricultural fields and roads, which is an activity that is classified as high intensity [42].

It has been observed that PA has decreased in different populations due to changes in lifestyle [1, 17]. Transportation in vehicles such as cars or motorcycles is common in the Yaqui communities, which reduces physical activity. Likewise, a significant proportion of Yaqui adults are employed in maquiladora companies where, in addition to being a sedentary activity, they have to be transported for approximately an hour and a half in trucks, which further contributes to a more sedentary lifestyle. Despite this situation, the Yaquis are in a transition process that allows them to still be protected from a BMI increase by the practice of vigorous PA. This finding coincides with what has been reported in other studies [17, 61] and with some recommendations that indicate that to prevent weight gain moderate and vigorous physical activities should be combined [63, 64]. This information should be considered in health sector programs to encourage an increase in physical activity for the prevention and control of obesity in this Indigenous group.

A limitation of this study is the cross-sectional nature, which excludes the ability to distinguish cause from effect; however, the results are consistent with those found in different populations. One of the strengths of this study is that it provides information on different sociodemographic, anthropometric and lifestyle characteristics related to the health of a representative sample of Yaqui Indigenous people residing in their eight traditional villages. In addition, this is one of the few current studies that assessed the prevalence of obesity in an Indigenous group considering a representative sample and a probabilistic sample. This study also evaluated the factors associated with obesity, specifically for an Indigenous group. Another important feature is that the instruments used to assess the factors associated with obesity were designed exclusively for the study population.

## Conclusions

In conclusion, the prevalence of overweight, obesity and central obesity in Yaqui adults is high, similar to the prevalence found in the Mexican adult population. Higher values of the modernization index were associated with a higher BMI, while a greater consumption of a “prudent” dietary pattern and greater amount of vigorous physical activity were associated with a lower BMI. The evidence presented in this study suggests that interventions are needed and more research is required to determine the appropriate components of such interventions, in order to meet the needs of the Yaqui people.

## Data Availability

The datasets used and/or analyzed during the current study are available from the corresponding author on reasonable request.

## Abbreviations

BMI: Body mass index
CVD: Cardiovascular disease
ENSANUT MC: National health and nutrition survey of midway
FFQ: Food frequency questionnaire
MI: modernization index
NCD: Noncommunicable disease
PA: Physical activity
PC: principal component technique
SQ: sociodemographic questionnaire
T2D: Type 2 diabetes
